# Persistence of Root Exudates of *Sorghum bicolor* and *Solidago canadensis*: Impacts on Invasive and Native Species

**DOI:** 10.3390/plants13010058

**Published:** 2023-12-23

**Authors:** Muhammad Rahil Afzal, Misbah Naz, Raza Ullah, Daolin Du

**Affiliations:** 1 Jingjiang College, Jiangsu University, Zhenjiang 212013, China; 2 Institute of Environment and Ecology, School of the Environment and Safety Engineering, Jiangsu University, Zhenjiang 212013, China; misbahnaz.ray@yahoo.com; 3 Institute of Environmental and Agricultural Science, Faculty of Life Sciences, University of Okara, Okara 56130, Pakistan; raza1838@gmail.com; 4 School of Emergency Management, Jiangsu University, Zhenjiang 212013, China; 5 School of Agricultural Engineering, Jiangsu University, Zhenjiang 212013, China

**Keywords:** *Sorghum bicolor*, *Solidago canadensis*, root exudates, allelopathy, sorgoleone, legacy, invasive and native species

## Abstract

Root exudates of the invasive *Solidago canadensis* and the cereal crop *Sorghum bicolor* (L.) Moench cv. ‘Hybridsorgo’ were tested for allelopathic interactions against native and invasive plant species in a controlled environment. After the surface was sterilized, the seeds of two invasive species (*Bromus sterilis* and *Veronica persica*) and two native species (*Youngia japonica* and *Rumex acetosa*) were germinated and transplanted into the soil (1:1 mixture of coco peat and sand) that had been conditioned for one month by the cultivation of *Solidago canadensis* and *Sorghum bicolor*, both in combination or as unplanted controls. After an additional eight weeks of growth, morphometric measurements of the shoot and root, including foliar characteristics and above- and below-ground biomass accumulation, were performed. The results revealed significant inhibitory effects of root exudates released by *Sorghum bicolor* and *Solidago canadensis* on native species’ productivity and physiology. The invasive species exhibited variable growth responses, with *Veronica persica* showing reduced shoot and root expansion, but *Bromus sterilis* revealed increased shoot and root biomass allocation and nutrition under the exudate treatments. Exudates from *Solidago canadensis* and *Sorghum bicolor* together showed synergistic negative effects on native species, while they promoted growth and nutrition in *Veronica persica*. Taken together, the differential species responses indicate that the tested native species were more sensitive to the allelopathic compounds than the invasive species, which is in line with the theory of novel weapons. The legacy effects of root exudates of both *Sorghum bicolor* and *Solidago canadensis* could promote invasive establishment through imposing allelochemical interference competition against native plant species. Understanding the specific allelopathic mechanisms may help with the development of integrated strategies for managing invasive species.

## 1. Introduction

As an important ecological process, allelopathy can impact plant community dynamics and the establishment of invasive species [1,2]. Allelopathic substances released from living roots and their persistence in soils might provide certain invasive plants a competitive edge in colonizing new regions or suppressing native vegetation [3,4,5]. Two plant species, *Solidago canadensis* and *Sorghum bicolor*, have shown well-established allelopathic effects on other plants through root exudation [6,7,8,9].

*Sorghum bicolor*, an important cereal crop, has the strong ability to release allelopathic compounds from its roots that can inhibit the germination and growth of neighboring plants [4,10,11,12]. Sorgoleone has been identified as the main allelochemical exuded naturally from *Sorghum bicolor* roots and is a highly phytotoxic compound reported to inhibit root and shoot growth of test plant species at IC50 value under 10 μM [12,13,14,15,16]. The secretion of sorgoleone from *Sorghum bicolor* roots appears to occur independently of plasma membrane H^+^-ATPase activity and proton gradients [17]. This uncoupling from proton motive forces suggests vesicular transport may mediate sorgoleone exudation to avoid autotoxicity. Similar to many phenylpropane and flavonoids, sorgoleone is likely synthesized on the surface of the endoplasmic reticulum and then transported from its site of synthesis to the extracellular space through vesicles [18,19]. Several studies have shown that sorgoleone is a potent photosystem II inhibitor that inhibits photosynthesis and reduces the chlorophyll content in treated plants by hindering the oxidation of the plastoquinone pool in photosystem II [20,21]. Sorgoleone exhibits pre-emergence herbicidal activity by inhibiting mitochondrial respiration in germinating seeds and seedlings [22,23,24]. Furthermore, sorgoleone can alter the soil microbial community, for example, limiting root colonization of *arbuscular mycorrhizal* fungi, which, in turn, may affect plant growth [25,26]. However, it has been found that certain plant species respond differently to allelochemicals. For example, the weed species *Sorghum halepense* exhibited a higher tolerance towards sorgoleone compared to crop plants and certain native grasses [9,27,28]. This indicates the natural variation in the allelochemical sensitivity of different plant species. Notably, throughout the growing season, living *Sorghum bicolor* roots continuously exude sorgoleone for weeks. Sorgoleone can persist in soil environments for prolonged durations after exudation, contingent upon various factors, including soil type, moisture content, temperature, and microbial activity [29,30,31]. Research has documented the presence of residual soil sorgoleone in unplanted *Sorghum bicolor* fields for 16–20 weeks following harvest [9,21,32,33]. This persistence of sorgoleone enables it to operate as a post-emergent and pre-emergent herbicide in *Sorghum bicolor* agroecosystems, inhibiting the growth of both annual and perennial weeds through residual soil activity.

The allelopathic potential of *Sorghum bicolor* has several implications in agriculture. Herbicide use can be minimized by rotating *Sorghum bicolor* with cereals, legumes, and vegetables to suppress weed development in succeeding crops [9,34,35,36,37]. For example, sorghum–wheat, sorghum–soybean, and sorghum–corn rotations in the Midwestern United States decreased weed pressure in the following crops by 21–51% compared to continuous cropping of the same species [38]. The weed-suppressing effects of *Sorghum bicolor* sorghum rotation can persist for multiple growing seasons. In Kansas, USA, sowing *Sorghum bicolor* in the prior year resulted in 54% lower weed density and 61% lower biomass in wheat compared to continuous wheat [39]. *Sorghum bicolor* as a mulch or as a cover crop can also inhibit weed growth in agricultural production systems through sorgoleone release and leaching following the gradual breakdown of *Sorghum bicolor* residues [4,9,40,41]. For instance, when used as a summer smother crop in broccoli farming, a sorghum–sudangrass hybrid cover crop decreased weed biomass in the subsequent broccoli crop by 72% compared to fallow controls [42]. Field trials of sorgoleone formulations exhibited effective control of broadleaf and grass weeds at rates under 2 kg/ha [43]. Following the recognition of sorgoleone as a natural herbicide, efforts have spurred the formulation of *Sorghum bicolor* root exudates as a commercial bioherbicide product [23,32].

*Solidago canadensis* is a perennial herbaceous plant native to North America and considered invasive in Europe, Asia, and Oceania. It has been reported that *Solidago canadensis* roots produce multiple phenolic acids, including ferulic acid, caffeic acid, and p-coumaric acid, which, after release into the rhizosphere, affect other plants through allelopathic activity [44,45]. In test plant species, caffeic acid, in particular, inhibits seed germination, root elongation, and seedling development. Its possible modes of action include disruption of cell division, mitochondrial respiration, plasma membrane integrity, and hormonal signaling pathways [46,47]. *Solidago canadensis* leaf and root extracts reduced conifer seed germination and seedling growth in controlled trials [7,48,49]. According to Abhilasha et al. [50], the germination of pine and spruce seeds exposed to *Solidago canadensis* extracts was entirely stopped by caffeine doses of 175–200 μM. Moreover, field surveys show negative relationships between *Solidago canadensis* cover and native tree seedling density [51]. For example, in New England forests, native tree seedling density decreased exponentially from 5000–6000/ha to less than 1000/ha as *Solidago canadensis* cover increased from 1% to 60% [52]. Therefore, allelochemicals from *Solidago canadensis* may limit native tree regrowth where invaded. There is also evidence that *Solidago canadensis* residues inhibited carrots, alfalfa, timothy, and barley biomass mainly due to high soil ferulic acid concentrations [53,54], highlighting *Solidago canadensis* allelopathic effects on diverse plant species. Apart from caffeic acid and ferulic acids, *Solidago canadensis* roots produce flavonoid quercetin, which has been reported to show phytotoxic effects in lab studies [50]. The diversity of allelopathic chemicals found in *Solidago canadensis* indicates that the plant uses a number of inhibitory pathways to obstruct physiological processes in plants to obtain an edge over competitors. *Solidago canadensis* has an established root system that can maintain a continuous release of allelochemicals into the soil. It is likely that residual allelochemicals accumulate over time in *Solidago canadensis* growth zones.

Even though many studies have evaluated the phytotoxicity of *Sorghum bicolor* and *Solidago canadensis* root exudates on model plants and common crops, research comparing the allelopathic effects of compounds found in *Solidago canadensis* and *Sorghum bicolor* roots on native versus invasive species is still limited. Therefore, this study aimed to determine the residual allelopathic effects of root exudates from *Sorghum bicolor* and *Solidago canadensis* on different invasive and native plant species. The invasive species *Veronica persica* (common names: Persian speedwell and bird’s-eye speedwell) is native to Europe and parts of Asia but has spread globally as an aggressive weed [55]. It infests croplands, nurseries, lawns, and natural areas across North America, South America, Australia, Africa, and New Zealand [56,57,58]. *Veronica persica* competes strongly with crops and native vegetation, reducing yields by up to 45% in cereals and 79% in legumes [59]. Its rapid growth allows it to quickly colonize open habitats. In Australia, *Veronica persica* has invaded over 25 million acres of cropland and caused estimated annual losses of AUD 15 million [28,60]. Other countries likely incur comparable agricultural costs for *Veronica persica* management [61]. The invasive grass species *Bromus sterilis* (barren brome) is native to the Mediterranean region but has invaded ecosystems worldwide, including North and South America, Australia, and South Africa [62]. It colonizes roadsides, crop fields, rangelands, pine forests, and coastal habitats [63]. *Bromus sterilis* infestations replace native plants, increase soil erosion, and reduce livestock forage quality [64]. In western North America, it dominates more than 10 million acres of grasslands and shrublands [65]. Dense stands exacerbate wildfire risk, and post-fire re-establishment hinders native community recovery [66]. Annual economic impacts of *Bromus sterilis* in the western United States alone are estimated at over USD 50 million from both losses in livestock production and control costs [67]. Both species reproduce prolifically by seeds. *Veronica persica* produces up to 2500 seeds per plant under ideal conditions [68]. *Bromus sterilis* forms persistent soil seed banks averaging 3800 seeds/m^2^ in invaded California grasslands, allowing populations to recover after control efforts [69]. Their adaptive biology and substantial ecological impacts demonstrate the need for sustainable integrated management techniques such as allelopathic cover crop rotations.

We hypothesized that the legacy of residual root exudates in the soil would have an overall inhibitory effect on both invasive and native plant growth and performance. However, we predict invasive species may show greater tolerance to the allelopathic compounds compared to native species. Understanding these interactions could provide insights into the establishment and management of invasive plants as well as the ecological implications of allelopathic crop cultivation.

## 2. Results

### 2.1. Above-Ground Growth Responses of Invasive and Native Species to Sorghum bicolor and Solidago canadensis Root Exudates

**Figure 1:**
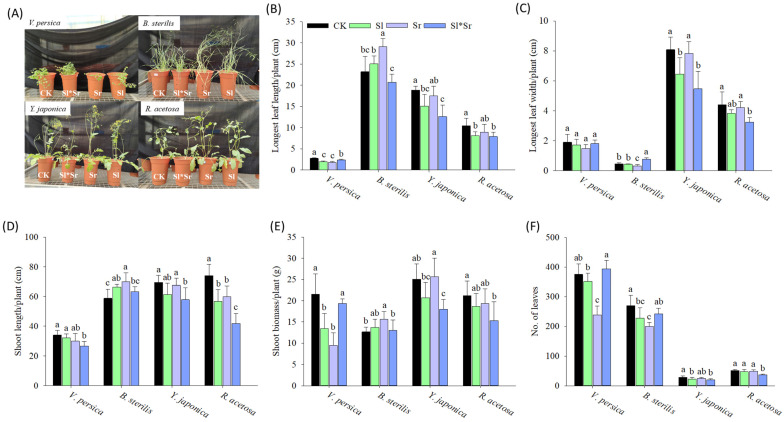
Effect of root exudates of *Sorghum bicolor* and *Solidago canadensis* on above-ground growth responses: (**A**) phenotype of 8-week-old plants, (**B**) longest leaf length, (**C**) longest leaf width, (**D**) shoot length, (**E**) shoot biomass, (**F**) number of leaves of invasive (*B. sterilis* and *V. persica*) and native (*Y. japonica*, and *R. acetosa*) species. CK, Sl, Sr, and Sl*Sr in the graphs represent the treatments: Control (no donor plants), *Solidago canadensis* alone, *Sorghum bicolor* alone, and *Solidago canadensis* + *Sorghum bicolor* intercropped, respectively. Bars show means ± SD (*n* = 5). Different letters above bars indicate significant differences between treatments for each species and trait at *p* ≤ 0.05 by LSD test.

The results indicate a significant impact of root exudates of *Sorghum bicolor* and *Solidago canadensis* on most of the above-ground growth traits of both invasive and native plant species, but the effects differ by species (Figure 1). Among the invasive species, *Veronica persica* showed a significantly decreasing trend in its longest leaf length, shoot length, and shoot biomass in response to all allelopathy combinations compared to the control (Figure 1B,D,E). However, there was no significant impact observed on the longest leaf width (Figure 1C). For invasive *Bromus sterilis*, shoot biomass, shoot length, and longest leaf length increased with allelopathy except for longest leaf width, which only increased with combined *Sorghum bicolor* and *Solidago canadensis* exudates. In addition, both the invasive species showed a decrease in the number of leaves in all allelopathy combinations (Figure 1F). Among all the allelopathy combinations, *Sorghum bicolor* alone had the strongest impact on invasive species. On the other hand, native *Youngia japonica* and *Rumex acetosa* showed reduced shoot biomass, root biomass, longest leaf length, longest leaf width, and the number of leaves when exposed to *Sorghum bicolor* alone, *Solidago canadensis* alone, or both allelopathy. Both the native species showed the highest sensitivity to the combined *Sorghum bicolor* and *Solidago canadensis* allelopathy.

### 2.2. Below-Ground Growth Responses of Invasive and Native Species to Sorghum bicolor and Solidago canadensis Root Exudates

**Figure 2:**
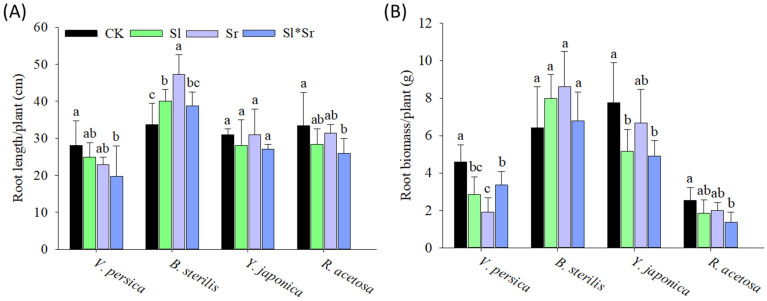
Effect of *Sorghum bicolor* and *Solidago canadensis* root exudates on below-ground growth responses: (**A**) root length and (**B**) root biomass of invasive (*B. sterilis* and *V. persica*) and native (*Y. japonica*, and *R. acetosa*) species. CK, Sl, Sr, and Sl*Sr in the graphs represent the treatments: Control (no donor plants), *Solidago canadensis* alone, *Sorghum bicolor* alone, and *Solidago canadensis* + *Sorghum bicolor* intercropped, respectively. Bars show means ± SD (*n* = 5). Different letters above bars indicate significant differences between treatments for each species and trait at *p* ≤ 0.05 by LSD test.

The root exudates of *Sorghum bicolor* and *Solidago canadensis* revealed significant allelopathic effects on the root systems of both invasive and native species, but the sensitivity varied. The root exudates significantly reduced the root length and root biomass of invasive *Veronica persica* across all treatments (Figure 2). However, combined *Sorghum bicolor* and *Solidago canadensis* had a higher inhibiting effect on root length but not on root biomass than *Solidago canadensis* alone and *Sorghum bicolor* alone compared to the control. *Sorghum bicolor* alone root exudates overall decreased root length and root biomass more than *Solidago canadensis* alone, but the impact on root length was non-significant. For invasive *Bromus sterilis*, all allelopathy combinations increased root length and root biomass, with *Sorghum bicolor* alone having the greatest effect. On the other hand, native *Youngia japonica* had a slightly reduced root length but a significantly decreased root biomass in response to *Solidago canadensis* alone and combined *Solidago canadensis* and *Sorghum bicolor* root exudates than *Sorghum bicolor* alone compared to the control (Figure 2). Likewise, native *Rumex acetosa* showed a decrease in root length and root biomass across all the allelopathy treatments, but the combined *Solidago canadensis* and *Sorghum bicolor* allelopathy gave the strongest reductions in both measures.

### 2.3. Responses of Invasive and Native Species to Sorghum bicolor and Solidago canadensis Root Exudates in Physiological Traits

**Figure 3:**
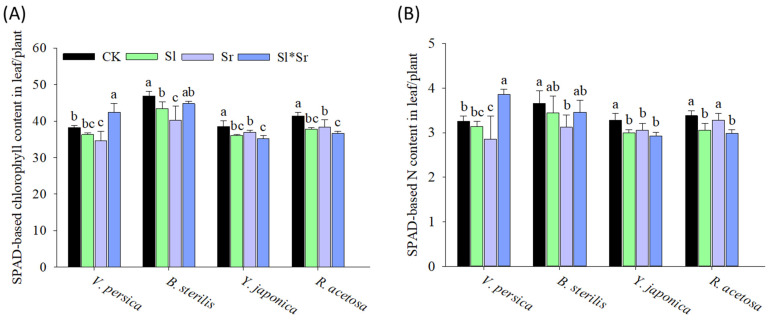
Effect of *Sorghum bicolor* and *Solidago canadensis* root exudates on (**A**) leaf nitrogen and (**B**) chlorophyll content of invasive (*B. sterilis* and *V. persica*) and native (*Y. japonica* and *R. acetosa*) species. CK, Sl, Sr, and Sl*Sr in the graphs represent the treatments: Control (no donor plants), *Solidago canadensis* alone, *Sorghum bicolor* alone, and *Solidago canadensis* + *Sorghum bicolor* intercropped, respectively. Bars show means ± SD (*n* = 5). Different letters above bars indicate significant differences between treatments for each species and trait at *p* ≤ 0.05 by LSD test.

In terms of physiological indicators in invasive *Veronica persica*, *Solidago canadensis* alone and *Sorghum bicolor* alone significantly reduced nitrogen content and chlorophyll content compared to the control. However, the combined *Solidago canadensis* and *Sorghum bicolor* resulted in a significant increase in both indicators (Figure 3). For invasive *Bromus sterilis*, all the treatments reduced leaf nitrogen content and chlorophyll content with *Sorghum bicolor* alone, having maximum effect compared to the control. The native species *Youngia japonica* and *Rumex acetosa* had lowered leaf nitrogen content and chlorophyll content in response to all treatments compared to the control.

## 3. Discussion

The findings of the current study suggest that root exudates produced by *Solidago canadensis* and *Sorghum bicolor* had strong allelopathic effects and significantly affected the growth and physiology of the tested native and invasive plant species. The effects vary, contingent on the plant species and the formulation (single or combination) utilized. Our results demonstrate contrasting growth responses of invasive *Veronica persica* and *Bromus sterilis* to the root exudate treatments. *Veronica persica* exhibited a decrease in the shoot growth traits, including biomass, length, and longest leaf length, when exposed to all exudate treatments compared to the control (Figure 1). This indicates a higher susceptibility of *Veronica persica* to the allelochemicals [70,71]. Previous studies have also reported the inhibitory effects of *Sorghum bicolor* and other crop residues on the growth of *Veronica persica*, which was attributed to the release of phenolic acids and other allelochemicals [72,73]. On the other hand, it was observed that the shoot growth of *Bromus sterilis* exhibited an increase when subjected to the exudate treatments except for leaf width (Figure 1). This finding implies the possibility of growth promotion by hormesis, particularly at low concentrations of allelochemicals [74]. Previous studies have established the phenomenon of hormetic growth stimulation of invasive species through allelopathy [75,76,77]. Further, all the treatments, especially combined exudates, caused a reduction in root growth (root length and biomass), indicating root inhibition (Figure 2), whereas *Bromus sterilis* exhibited an increased root growth under all the treatments, which once again suggests the potential hormesis. Other studies also found differential root growth effects of allelopathy contingent upon the invader species [78]. Varying shoot and root growth responses of *Veronica persica* and *Bromus sterilis* reveal the different allelochemical sensitivities of these invasive species. The decreases in the leaf number observed for both invasive species across all treatments suggest the possible inhibition of leaf initiation and expansion [79]. In terms of physiology, the results revealed variable effects on nitrogen and chlorophyll content in *Veronica persica*, decreasing under individual exudates while increasing under combined exudates (Figure 3). This implies that mixed exudates may have complementary impacts on photosynthesis and nutrition [80,81]. On the contrary, the observed decreases in nitrogen and chlorophyll for *Bromus sterilis* point to decreased nutrition and photochemistry as a result of allelopathy [82,83]. Overall, the findings demonstrate the species-specific physiological responses of invasive species to allelopathy.

On the contrary to the varied responses of invasive species, the root exudate treatments resulted in diminished shoot growth, root development, leaf size, and leaf number in both native species *Youngia japonica* and *Rumex acetosa* (Figure 1 and Figure 2). This shows the sensitivity of the native species to the allelopathy. The observed decrease in leaf nitrogen and chlorophyll content is indicative of imbalanced nutritional status and decreased photosynthetic activity. The significant decrease in growth under mixed *Sorghum bicolor* and *Solidago canadensis* exudates indicates the synergistic allelopathic effects, as previously reported for native species [8]. Overall, our findings indicate that the tested native species showed more sensitivity to the allelochemicals than the invasive species. This supports the novel weapons hypothesis, where invasive species exhibit greater allelopathic resistance than native species [84]. The decreased growth and altered nutrition of native species under the persistent root exudate treatments suggest the potential for allelopathy to assist invasion by suppressing native competitors. While differential inhibitory effects were observed across plant species, the tested native species generally exhibited more restricted growth metrics under root exudate conditions compared to the invasive species. However, taxonomic differences within groups may play a role in these patterns, along with invasion status, emphasizing the need for controlled within-family assessments.

It is probable that the persistent root exudates induced allelopathic effects via multiple mechanisms. Documented allelochemicals in *Sorghum bicolor* include benzoic, p-hydroxybenzoic, vanillic, ferulic, chlorogenic, m-coumaric, p-coumaric, momilactones, gallic, and caffeic acids [85], p-hydroxybenzaldehyde, dhurrin, sorgoleone [16,86], m-hydroxybenzoic acid, and protocatechuic acid [87] with the potential to reduce weed growth. These compounds can inhibit growth by affecting cell division, nutrient uptake, photosynthesis, and hormone balance [88,89]. For instance, momilactones may act as auxin inhibitors, whereas polyacetylenes can inhibit mitochondrial respiration [90]. The generation of reactive oxygen species leading to oxidative damage is another potential mode of action [91]. Sorgoleone can inhibit photosynthesis by blocking electron transport in photosystem II [24]. The sorgoleone concentrations achieved in the root exudate treatments, even after dilution in the soil, were likely sufficient to disrupt photosystem II function in sensitive native species, contributing to the measured reductions in the chlorophyll content and growth (Figure 1, Figure 2 and Figure 3). Sorgoleone also inhibits mitochondrial respiration [24], which may explain the stunted root growth patterns (Figure 2). For *Solidago canadensis*, compounds like caffeic acid can affect mitochondrial respiration, cell division, membrane integrity, and hormonal signaling [50,92]. The inhibition of leaf initiation and expansion in native species indicates possible interference with cell division and hormone pathways governing growth processes. The observed synergistic effects in the presence of combined exudates indicate that allelochemical compounds originating from both donor species may have exerted greater effects on shared physiological targets via additive or multiplicative mechanisms of action [83]. The varying species responses indicate how allelopathy effects depend on the interacting allelochemical–receiver combination and concentration-dependent hormetic effects [83]. Further investigation is required to identify specific allelochemicals and their mechanisms involved in inducing the growth and physiological changes observed here.

The results have several implications for managing plant invasions. First, the findings highlight the feasibility of using *Sorghum bicolor* as a cover crop where its allelopathic effects can suppress invasive and encourage native plants through residual root exudates. Allelopathic rotations can provide longer-term and environmentally friendly weed control than mowing or herbicides alone [4]. The stronger growth inhibition of native species points to potential trade-offs, but lower exudate doses could be optimized to reduce native plant suppression while still keeping the invader species at a minimum. Second, the results suggest residual *Solidago canadensis* exudates could help limit certain invaders like *Veronica persica* through allelopathic interference. Fostering competitive invasive species like *Solidago canadensis* could aid invader resistance without requiring chemical control [2,93]. Lastly, the results provide baseline data on species-specific allelopathy responses that can inform integrated management programs relying on multiple control techniques.

## 4. Materials and Methods

### 4.1. Experimental Material

Seeds of two invasive species, *Bromus sterilis* and *Veronica persica*, and two native species, *Youngia japonica* and *Rumex acetosa*, were collected in April 2022 from a field site at Xianglushan (119°13′41′′ E, 32°07′42.9′′ N) around Zhenjiang in Jiangsu Province, China. Seeds were stored in paper bags at room temperature until their experimental use in August 2022.

### 4.2. Seed Germination

Seeds of four species were surface sterilized in 10% hydrogen peroxide for 10 min, rinsed thoroughly with distilled water, and germinated in plastic germination trays (25 cm × 15 cm) filled with coco peat growth medium. Trays were kept in a germination chamber at 25 °C/15 °C day/night temperatures and a 12 h photoperiod for 2 weeks until seedlings emerged.

### 4.3. Donor Species Growth

Sorghum *Sorghum bicolor* (L.) Moench cv. ‘Hybridsorgo’ and goldenrod (*Solidago canadensis*) plants were grown from seed in plastic pots (15 cm diameter, 20 cm height) filled with a 1:1 mixture of coco peat and sand. Five seeds of each donor species were directly sown in each pot and thinned to one plant per pot after 2 weeks. Donor plants were grown in a glasshouse for 4 weeks at 25 °C/15 °C day/night temperatures and ambient light conditions. The pots were watered daily to maintain optimal soil moisture.

### 4.4. Treatments

After 4 weeks of growth, the donor plants were uprooted, and the soil containing the root exudates was removed from each pot. The soil from pots of the same treatment was mixed thoroughly and used to refill the pots. This ensured even distribution of the root exudates for the following treatments applied with 5 replications per treatment: 1. control (no donor plants), 2. *Solidago canadensis* alone, 3. *Sorghum bicolor* alone, and 4. *Sorghum bicolor* + *Solidago canadensis* intercropped. The 2-week-old germinated seedlings of the invasive and native species with uniform phenotypes were transplanted into the pots containing the donor species’ root exudates. Each pot contained one seedling of each receiver species. Seedlings were watered daily and grown for 8 weeks before harvest.

### 4.5. Growth Trait Measurement

The following growth parameters were measured on each plant at harvest: number of leaves, length of the longest leaf (cm), width of the longest leaf (cm), shoot height (cm), root length (cm), leaf nitrogen content, relative chlorophyll content, aboveground biomass (g), and belowground biomass (g). Plant height and root length were measured with a ruler, and the relative chlorophyll content and leaf nitrogen content were measured with a SPAD-502 chlorophyll content analyzer.

### 4.6. Data Analysis

All the experimental data were pooled for calculations of means and ± SD and analyzed by one-way ANOVA, followed by the LSD test at *p* ≤ 0.05 to determine the statistical significance of the differences between individual treatments, indicated by letters above bars.

## 5. Conclusions

Our results revealed that root exudates from *Sorghum bicolor* and *Solidago canadensis* exerted strong phytotoxic effects in tested plant species. Invasive species showed variable sensitivities, with *Veronica persica* exhibiting susceptibility and *Bromus sterilis* demonstrating tolerance. The native species *Youngia japonica* and *Rumex acetosa* experienced consistent growth and performance inhibitions under root exudate treatments. While native species appeared more negatively impacted overall, taxonomic differences between the evaluation groups confound definitive conclusions regarding the intrinsic effects of invasive status. Therefore, comparing more closely related taxa within the same families could better isolate invasion-associated traits from phylogenetic variability. Still, certain aggressive invaders like *Veronica persica* proved vulnerable to allelochemical legacy impacts in soil. Optimizing the cultivation and rotation of bioactive cover crops could provide ecological weed control options. Species-specific allelopathic responses underscore the need for integrated management deploying multiple approaches against heterogeneous exotic plant populations. Further controlled experiments on taxonomically paired invasive and native species can help elucidate mechanisms governing allelopathic resistance. Building on this foundational study, elucidating why some invasive plants tolerate phytotoxins could inform protection strategies leveraging plant–plant inhibitory interactions to impede exotic plant ingress while recovering native biodiversity.

## Data Availability

There is no data availability statement.
